# Incidence of *kelch13* and *Pfmdr1* gene mutations associated with antimalarial drug resistance in *Plasmodium falciparum* isolates from Ethiopia: a systematic review and meta-analysis

**DOI:** 10.1186/s12879-026-13164-2

**Published:** 2026-03-26

**Authors:** Temesgen Mitiku Yeshanew, Bokretsion Gidey Brhane, Betelhem Abebe Begashew, Gemechis Waktole Bayisa, Nega Birhane

**Affiliations:** 1 Department of Biotechnology, Dambi Dollo University, Dambi Dollo, Ethiopia; 2 Department of Medical Biotechnology, Institute of Biotechnology, University of Gondar, Gondar, Ethiopia; 3 Ethiopia Public Health institute, Addis Ababa, Ethiopia

**Keywords:** Malaria, Resistance, *Pfmdr1*, Kelch13, *Plasmodium falciparum*, Mutation

## Abstract

**Background:**

The increasing prevalence of drug-resistant *Plasmodium* parasites, driven by factors including genetic mutations, intensifies the challenge of malaria eradication. This meta-analysis focused on the genetic component of resistance, specifically evaluating mutations in the *kelch13* (R622I, P441L and A675V) and *Pfmdr1* (Y184F and D1246Y) genes within Ethiopian Plasmodium falciparum strains.

**Methods:**

A systematic literature search was conducted across the PubMed/MEDLINE, Scopus, Web of Science, Cochrane Library, and Google Scholar databases. Eligible studies included primary data on the prevalence of *kelch13* and *Pfmdr1* gene mutations in *Plasmodium falciparum* isolates from Ethiopia. Two reviewers independently conducted study selection, data extraction, and quality assessment. Heterogeneity was evaluated using the I² statistic, and a random-effects model was utilized to pool prevalence estimates with 95% confidence intervals (CI). Subgroup and sensitivity analyses were performed to explore sources of heterogeneity. Publication bias was assessed using funnel plots and Eggers test. All analyses were carried out using STATA software version 16.

**Results:**

A total of fifteen studies were reviewed and analyzed, comprising 11,269 samples and 5,521 positive cases of *Plasmodium falciparum*. Pooled prevalence of *kelch13* gene mutations at R622I, A675V, and P441L was 11%, 1% and 0% respectively. Pooled prevalence of *Pfmdr1* gene mutations at D1246Y and Y184F was 6% and 77%, respectively. Subgroup analysis revealed this resistance marker has significantly increased over time, rising from 66% (2007–2014) to a concerning 91% (2015–2025).

**Conclusions:**

While most resistance markers remain rare or absent, the significant rise in the *Pfmdr1* Y184F mutation highlights a growing concern regarding antimalarial drug resistance in Ethiopia.

## Background

 Malaria is a lethal infectious illness that remains prevalent and has a significant mortality rate, especially in tropical and subtropical areas [1, 2]. Human malaria is caused by five different *Plasmodium* species, with *Plasmodium falciparum* being the most predominant and deadly. Additionally, *Plasmodium vivax* contributes significantly to global illness and death [3, 4]. The World Health Organization (WHO) reported that in 2023, there were 263 million malaria infections and 597,000 malaria-related deaths globally. More than 95% of these mortalities occurred in the African region, as defined by the WHO [5].

Ethiopia ranks as the fourth-largest malaria-endemic country in Africa, with approximately 9,560,000 (3.6%) cases documented in 2023. Over the last two years, Ethiopia has faced numerous challenges, including the insufficient implementation of malaria prevention and control strategies in conflict affected areas, the emergence of the new vector *Anopheles stephensi*, vector resistance to insecticides, and the impacts of climate change and variability [5].

One of the fundamental components of malaria control is the utilization of antimalarial drugs, which are critical for alleviating the burden of malaria. Since 2004, Artemether-lumefantrine has been the first-line treatment for uncomplicated *Plasmodium falciparum* malaria in Ethiopia [6]. However, the effectiveness of drugs can be partly affected by genetic differences in the genes that encode the enzymes involved in drug metabolism [7]. 80% of clinically administered antimalarial drugs designed to target *Plasmodium falciparum* are metabolized by cytochrome P450 enzymes in the liver, particularly in phase I metabolic reactions [8, 9]. CYP1A2, CYP2A6, CYP2B6, CYP2C8, CYP2C9, CYP2C19, CYP2D6, CYP2E1, CYP3A4, and CYP3A5 are the main isoforms that metabolize drugs [10, 11]. Among the cytochrome P450 genes, CYP2C8 is responsible for the metabolism of amodiaquine (AQ) [12], whereas both CYP2C8 and CYP3A4 are involved in the metabolism of chloroquine [13].

Nevertheless, the emergence of drug resistance in *Plasmodium falciparum* poses a significant threat to these initiatives, as the parasite has developed resistance to even the modern generation of the most effective antimalarial medications [14, 15]. The primary factors contributing to the development of drug-resistant strains of malaria include genetic influences [16], erythrocyte interactions [17], and coinfections with other diseases [18]. The increase in drug-resistant strains compromises treatment effectiveness, resulting in prolonged illness periods, elevated mortality rates, and increased healthcare costs [17].

Single-nucleotide polymorphisms (SNPs) have been discovered in various genes of *Plasmodium falciparum* that are correlated with resistance to antimalarial medications, such as Artemisinin derivatives (ARTs), chloroquine (CQ), and sulfadoxine-pyrimethamine (SP) [19]. In general, Artemisinin resistance has been concentrated along the Thai‒Cambodian border, a known epicenter for multidrug-resistant malaria parasites [20]. ARTs are initially activated by heme and iron, leading to damage to proteins and the proteasome. This damage results in prolonged cellular stress and ultimately contributes to parasite death [21]. Mutations in *Pfkelch13* are strongly responsible for resistance to the Artemisinin class of antimalarial drugs. Currently, more than 200 SNPs have been formally associated to Artemisinin resistance (ART-R) within the *Pfk13* gene. Nevertheless, only 13 of these SNPs have been conclusively associated to ART-R: F446I, N458Y, M476I, Y493H, R539T, I543T, P553L, R561H, P574L, C580Y, R622I, C469Y, and A675V [22]. A study revealed that P441L, P574L, and A675V, has been identified in Ethiopia [23], Eritrea [24], and Rwanda [25].

The *Pfmdr1* gene in *Plasmodium falciparum* is associated with altered responses to ACT, especially those that include lumefantrine and amodiaquine [26, 27]. Mutations in codons 86, 130, 144, 184, 1034, 1042, 1226, and 1246 modify the charge and structure of the multidrug-resistant transporter (*Pfmdr1)* in the vacuolar compartment, resulting in enhanced drug elimination [26, 28]. The specific mutations N86Y, Y184F, and D1246Y have been documented in Ethiopia [23, 29, 30]. The amplification of the *Pfmdr1* gene is a significant resistance mechanism, with increased gene copies associated with diminished sensitivity to drugs such as lumefantrine and mefloquine [31].

The efficacy of treatment is directly linked to this genetic variation. For example, the failures observed in Artemisinin-based combination therapy (ACT) in Cambodia have been correlated with mutations in the *Pfmdr1* gene [32]. The incidence of *Pfmdr1* gene mutation differs widely, with reported figures such as 20% in Venezuela [33], 5.5% in Myanmar [34], 11% in Suriname [35], and 54.2% in Ethiopia [36]. To enhance malaria elimination efforts, the challenges mentioned above will be addressed by utilizing versatile genomic tools that enable cost-effective molecular surveillance of the disease. Among the common and versatile genomic tools used to detect SNPs, Molecular Inversion Probe (MIP) capture was first implemented in 2018 [37] and has since been used for the molecular surveillance of malaria, particularly in extensive nationwide surveys of drug resistance and population structure [38, 39]. This method employs short oligonucleotide probes that hybridize to and capture single-stranded DNA, forming circular regions of the genome [40]. Recent developments in sequencing technologies and computational analytical tools have improved the molecular surveillance of malaria. These advances enable the detection of drug resistance markers, tracking of malaria transmission, assessment of importation, and evaluation of the impact of interventions [41].

Despite the recognized importance of these genetic markers, evidence regarding the incidence of *kelch13* and *Pfmdr1* mutations in Ethiopia remains fragmented. Individual studies are often constrained by small sample sizes, specific geographic foci, or differing methodologies, making it challenging to obtain a reliable national estimate. Therefore, there is a critical need for a comprehensive synthesis of existing data to clarify the overall prevalence and spatiotemporal trends of these resistance-conferring mutations throughout the country.

## Methods

In accordance with PRISMA 2020 guidelines, a systematic review and meta-analysis was conducted to quantify the overall prevalence of the key drug resistance markers involving *kelch13* (R622I, P441L, and A675V) and *Pfmdr1* (Y184F and D1246Y) in *Plasmodium falciparum*, and to identify related factors in Ethiopia.

### Systematic review and meta-analysis question

What is the pooled prevalence of *kelch13* (R622I, P441L and A675V) and *Pfmdr1* (Y184F and D1246Y) gene mutation among *Plasmodium falciparum* parasites in Ethiopia?

### Systematic review and meta-analysis objectives

This systematic review and meta-analysis was aimed to determine the pooled prevalence of the *kelch13* (R622I, P441L and A675V) and *Pfmdr1* (Y184F and D1246Y) among *Plasmodium falciparum* parasites in Ethiopia.

### Registration of protocol

The systematic review and meta-analysis protocol has been registered in the International Prospective Register of Systematic Reviews (PROSPERO) ( https://www.crd.york.ac.uk/ CRD420251151712).

### Study area

The study took place in Ethiopia, a country with a total area of 1.1 million square kilometers. The country’s topography varies from 110 m below sea level to 4,550 m above sea level. The climate in Ethiopia is mainly tropical monsoon, with three distinct agroecological regions: lowlands, midlands, and highlands. In the highlands, the mean annual temperatures range from 10 °C to 16 °C, whereas in the midlands, they range from 16 °C to 29 °C. In the lowlands, the temperatures range from 23 °C to 33 °C. The annual rainfall in the highlands varies from 500 millimeters to over 2,000 millimeters, whereas in the lowlands, it ranges from 300 millimeters to 700 millimeters [42]. Currently, the Ethiopian population is estimated to be more than 120 million, with approximately 68% of the population living in malaria risk areas [43].

### Literature search strategy

This systematic review and meta-analysis was to determine the incidence of *kelch13* and *Pfmdr1* drug resistance genes in various regions of Ethiopia. Our search strategy, publication selection, and result reporting adhered to the PRISMA 2020 (Preferred Reporting Items for Systematic Reviews and Meta-Analyses) guidelines [44]. Comprehensive searches were conducted across PubMed/MEDLINE, Scopus, Web of Science, Cochrane Library, and Google Scholar databases. The combination rule we commonly used was [(“*Plasmodium falciparum*” OR “*P. falciparum*” OR “malaria”) AND (“prevalence” OR “epidemiology” OR “incidence” OR “frequency”) AND (“*Pfmdr1*” OR “*kelch13*” OR “*Pfk13*” OR “drug resistance”) AND “Ethiopia”]. Additionally, we manually searched Google and screened the reference lists of the included studies for more articles. Our search did not have a time restriction.

### Eligibility criteria

#### Inclusion criteria

This systematic review and meta-analysis included studies on the incidence of the *Plasmodium falciparum kelch13* (R622I, P441L, and A675V) and *Pfmdr1* (Y184F and D1246Y) genes published in various peer-reviewed journals. All included studies were original English research articles, encompassing all age groups, conducted between 2007 and 2025, and provided sufficient information on sample size and the prevalence of the *Plasmodium falciparum kelch13* (R622I, P441L, and A675V) and *Pfmdr1* (Y184F and D1246Y) genes. This information enabled an analysis of the overall prevalence of these genes in Ethiopia. Studies that utilized Illumina NextSeq, molecular inversion probes (MIP), Polymerase Chain Reaction-Restriction Fragment Length Polymorphism (PCR-RFLP), and High Resolution Melting (HRM) methods to detect mutations, as well as community-based studies conducted in Ethiopia, were considered.

#### Exclusion criteria

Of the studies identified, those that did not meet the pre-defined inclusion criteria for this systematic review and meta-analysis were excluded. This included any research not specifically focused on *Plasmodium falciparum* isolates from Ethiopia, as well as studies examining other *Plasmodium* species. Further exclusions were applied to research that did not investigate or report genotypic data on the *kelch13* or *Pfmdr1* genes, articles with unclear or unspecified methodologies for mutation detection, and studies from which quantitative data on mutation incidence or allele frequency could not be extracted. Additionally, secondary literature such as reviews, meta-analyses, letters, short communications, and conference abstracts was omitted. Laboratory-based studies that were not directly linked to patient-derived Ethiopian isolates including in vitro, ex vivo, or animal model research were also excluded, as were non-laboratory-based studies like surveys or clinical reports that lacked accompanying molecular genotyping results.

#### Article selection and data extraction

All the articles identified during the search were imported into EndNote 20 software and duplicate files were removed. The authors individually screened the articles on the basis of their titles, abstracts, and full texts to identify studies that potentially met the predetermined inclusion criteria. The authors subsequently developed a data extraction form via a Microsoft Excel spreadsheet and extracted the data from the full-text articles. The data extraction sheet included a large amount of information, such as the first author’s name, year of publication, region, study group, sample size, total number of positive findings, and prevalence percentage. Additionally, significant findings were qualitatively extracted for this systematic review and meta-analysis. Consistency checks were conducted on the extracted data by the two researchers (Temesgen Mitiku and Betelhem Abebe), and any inconsistencies were resolved through discussion.

#### Data synthesis and analysis

The necessary information was extracted from each original study via a Microsoft Excel spreadsheet. The data were exported to STATA version 16.1.0 for analysis using Meta package. For the first outcome variable, the prevalence and standard error of prevalence were generated for each study via the generate command in STATA. The pooled incidence is presented in a forest plot. To assess the degree of heterogeneity among the included studies, Cochran’s Q test (reported as the p value) and the index of heterogeneity (I^2^) were used. A statistically significant Cochran’s Q test result was considered when the p value was ≤ 0.05. The degree of statistical heterogeneity was interpreted via the I² statistic, with thresholds of 0% (none), 25% (low), 50% (moderate), and 75% (high) [45]. Owing to a high degree of heterogeneity, a random effects model was used to estimate the pooled prevalence of *kelch13* (R622I, P441L and A675V) and *Pfmdr1* (Y184F and D1246Y). Subgroup analysis using categorical data was conducted to explore potential differences between the years of the studies. A funnel plot was used to assess the presence of publication bias.

#### Procedural quality of the included studies

The included studies were independently assessed by five authors via the Joanna Briggs Institute (JBI) quality appraisal checklist for cross-sectional studies [46]. The assessment tool employs a set of criteria to evaluate the methodological quality of studies. These criteria encompass several domains: sample representativeness and recruitment, adequacy of sample size, description of subjects and setting, and completeness of sample coverage. The tool also examines the objectivity, standardization, and reliability of condition measurement. The final components of the evaluation include the appropriateness of statistical analysis and the adequate identification and accounting for confounders, subgroups, and differences, including the use of objective criteria for subpopulation identification. Cross-sectional studies were assessed using eight checklists. The resulting quality ratings were classified as high (above 75%), moderate (between 50% and 75%), or low (below 50%) (Table 1).

### Ethical consideration

This investigation was conducted entirely in accordance with PRISMA guidelines [47]. Approval from the institutional review board or ethics committee was not necessary because this was a systematic review and meta-analysis.

## Results

### Features of the studies

**Fig. 1:**
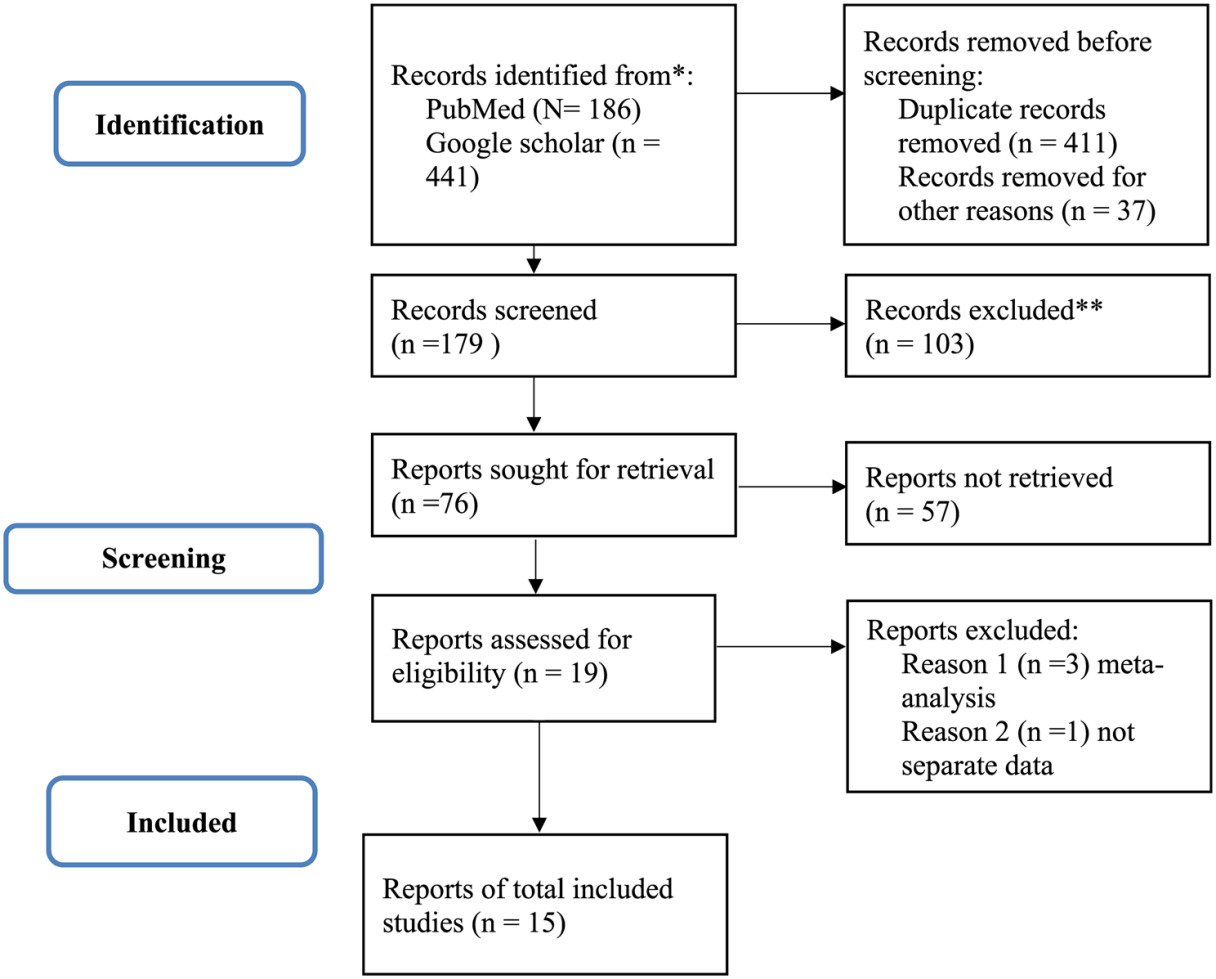
PRISMA flow diagram depicting study searching and selection history

The systematic identification process initially retrieved 627 records documenting the prevalence of *Plasmodium falciparum kelch13* (R622I, P441L, and A675V) and *Pfmdr1* (Y184F and D1246Y) gene polymorphisms in Ethiopia. Following the removal of 448 irrelevant or duplicate articles, the remaining 179 unique submissions underwent screening of titles and abstracts, leading to the exclusion of an additional 103 papers. The subsequent full-text eligibility review of the remaining 76 studies resulted in further exclusions: 57 papers were removed for lacking empirical data on the specified polymorphisms, and five were excluded as they represented systematic reviews or meta-analyses that did not report original, primary-level data. Ultimately, this rigorous screening and eligibility assessment process yielded fifteen eligible studies for inclusion in the final analysis (Fig. 1). The systematic review and meta-analysis included total 11,269 samples size and 5,521 *Plasmodium falciparum*-positive samples, which were successfully genotyped across fifteen studies [23, 29, 30, 48–59].

The majority of the research was conducted nationwide, with two study spanning seven regions: Afar, Amhara, Benishangul, Gambella, Oromia, SNNP, and Tigray [56] and Amhara, Tigray, Gambella, Afar, Somali, Oromia, and SNNPR [59]. Another study covered four of these regions: Afar, Amhara, Benishangul, and Gambella [23]. A third study was conducted in Afar, Gambella, Dire Dawa City and Oromia [57]. A fourth study focused on three regions: Amhara, Gambella, and Tigray [54]. Additionally, two studies were conducted in two regions, Amhara and Oromia [52, 55]. Three studies involved SNNP [29, 48, 49], two studies involved Oromia [50, 51], and two study involved the Amhara region [53, 58].

Most of the included studies were cross-sectional studies (73%) [23, 29, 30, 48–50, 52, 54–56, 59], while one was a retrospective study [51] and another was an in vivo study [53]. Sequencing was the most prevalent mutation detection method, employed in studies ( 73%) [23, 48, 49, 51–59], while two studies utilized PCR-RFLP [29, 50] and one used HRM [30].

**Table 1:** Methodological quality of the included studies

Reference	Selection				Compatibility	Exposure			Rating (high, moderate, low quality)
	Is the case definition adequate	Representativeness of the cases	Selection of controls	Definition of control		Ascertainment of exposure	Same method of ascertainment for cases and controls	Non– response rate	
1. [23].	*	*	*	*	*	*	*	*	High
2. [58].	*	*	*	*	*	*	*	*	High
3. [55].	*	*	*	*	*	*	*	*	High
4. [30].	*	*	*	*	*	*	*	*	High
5. [56].	*	*	*	*	*	*	*	*	High
6. [53].	*	*	*	*	*	*	*	*	High
7. [54].	*	*	*	*	*	*	*	*	High
8. [51].	*	*	*	*	*	*	*	*	High
9. [51].	*	*	*	*	*	*	*	*	High
10. [50].	*	*	*	*	*	*	*	*	High
11. [49].	*	*	*	*	*	*	*	*	High
12. [48].	*	*	*	*	*	*	*	*	High
13. [29].	*	*	*	*	*	*	*	*	High
14. [57].	*	*	*	*	*	*	*	*	High
15. [59].	*	*	*	*	*	*	*	*	High

**Table 2:** Description of individual study characteristics of the included studies

No	Author/Refer	Year of study	Region	Study design	Total sample size	Total PF. Positive	Method of detection	Y184F	D1246Y	R622I	P441L	A675V
1.	Mula et al., [29]	2007–2009	SNNP	cross-sectional	1,147	125	PCR-RFLP	ND	21/125	ND	ND	ND
2.	Brazeau et al., [48]	2007–2011	SNNP	Therapeutic Efficacy Studies	413	413	sequenced	63/413	95/413	0/413	0	0
3.	Mekonnen et al., [49]	2009–2011	SNNP	cross-sectional	410	195	sequencing	10/195	0/195	ND	ND	ND
4.	Heuchert et al., [50]	2013	Oromia	cross-sectional	338	171	PCR–RFLP	171/171	ND	ND	ND	ND
5.	Abera et al., [51]	2012–2013	Oromia	Retrospective	25	25	Whole-genome sequencing	25/25	0/25	ND	ND	ND
6.	Lo et al., [52]	2014	Amhara & Oromia	cross-sectional,	431	226	PCR & Sequencing	181/199	0/199	0	0	0
7.	Alemayehu et al., [53]	2017–2018	Amhara	in vivo	97	86	PCR & Capillary Sequencing	ND	ND	8/86	0	0
8.	Fola et al., [54]	2018–2021	Amhara, Gambella, Tigray	cross-sectional	920	609	MIP Sequencing & Deep Sequencing	504/609	57/609	49/609	ND	ND
9.	Tadele et al., [30]	2020	Benishangul-Gumuz	cross-sectional	230	225	Microscopy, PCR (varATS real-time PCR), and HRM	146/199	19/212	ND	ND	ND
10.	Holzschuh et al., [55]	2019–2020	Amhara and Oromia	cross-sectional	3,999	187	AmpSeq	176/181	ND	0 / 182	ND	ND
11.	Brhane et al., [23]	2019–2023	Amhara, Gambella, Oromia and Somali	cross-sectional	1,199	1,199	Illumina NextSeq and MIP sequencing	811/855	16/855	134/855	4/414	6/131
12.	Zeleke, et al.,[58]	2022–2023	Amhara	Cross-sectional	903	903	MIP & Whole-Genome Sequencing	803/830	0 / 830	369/833	ND	ND
13.	Jeang et al., [57]	2018–2022	Afar, Gambella, Dire Dawa City and Oromia	Surveillance	319	319	Sanger sequencing	ND	ND	4/72	ND	ND
14.	Letebo et al., [56]	2019–2023	Afar, Amhara, Benishangul, Gambella, Oromia, SNNP and Tigray	Cross-sectional	605	605	sequencing	503/538	0/605	57/572	6/571	10/572
15.	Kamaliddin [59]	2017–2022	Amhara, Tigray, Gambella, Afar, Somali, Oromia, and SNNPR	cross-sectional	233	233	Sequencing	ND	ND	31/44	ND	ND

ND: no detected

### Assessment of publication bias

**Fig. 2:**
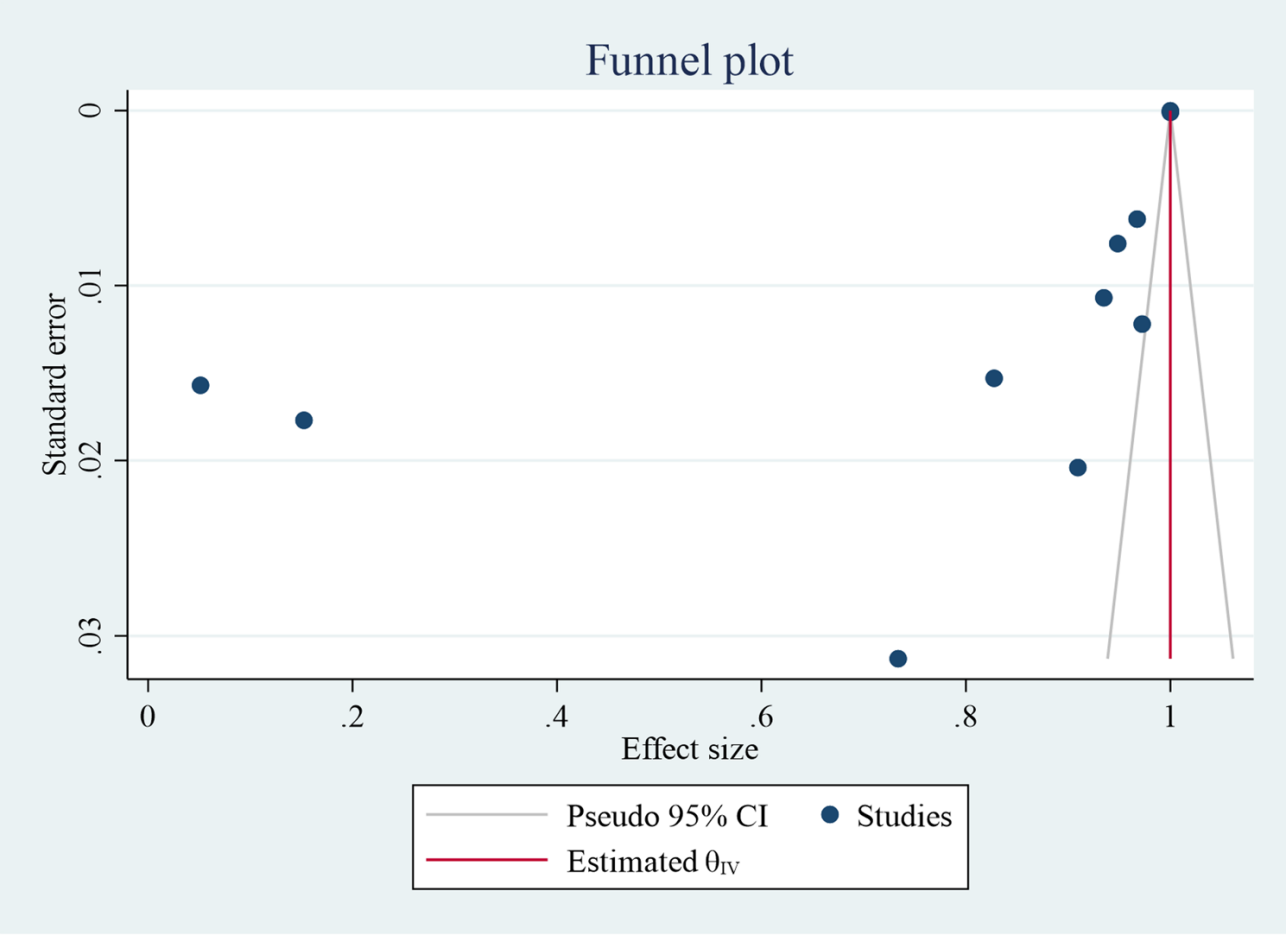
Funnel plot indicating the presence of publication bias

A funnel plot was used to visually assess the presence of publication bias. Each point on the funnel plots represents a unique study, and an uneven distribution reflects the presence of publication bias (Fig. 2). The symmetry assumption revealed publication bias (τ^2^ = 0.12) in the resistance pooled prevalence estimations.

### Pooled prevalence of *pfmdr1* Y184F mutations

**Fig. 3:**
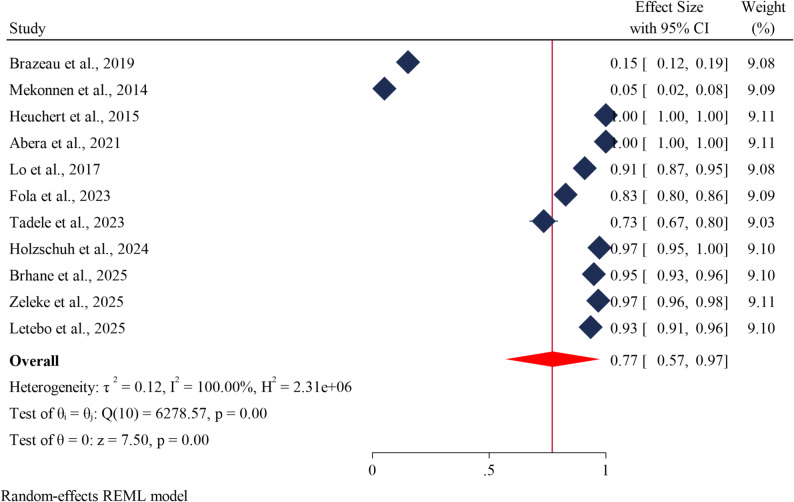
Forest plot showing the estimated prevalence of *Pfmdr1* drug resistance genes at codon Y184F in *Plasmodium falciparum* in Ethiopia: A systematic review and meta-analysis. 2025

By incorporating fifteen published research articles, we were able to estimate the prevalence of *Plasmodium falciparum Pfmdr1* drug resistance genes at codon Y184F in Ethiopia. The overall estimated pooled prevalence of *Plasmodium falciparum Pfmdr1* drug resistance genes at codon Y184F in Ethiopia was 77% (95% CI 57–97), and τ^2^ = 0.12 between-study heterogeneity (Fig. 3).

### *Pfmdr1* (Y184F) drug resistance genes subgroup analysis on the basis of study year

**Fig. 4:**
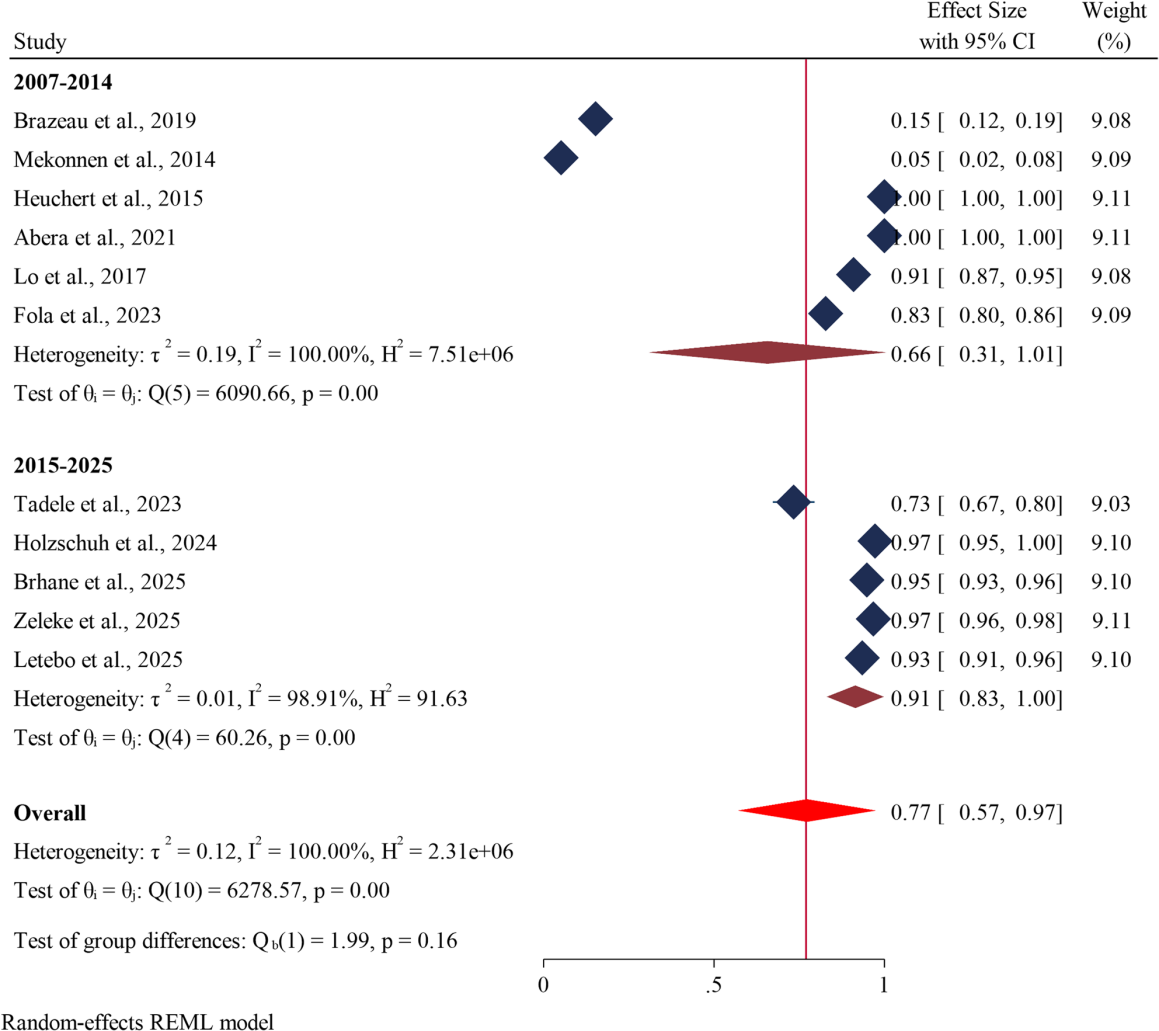
Forest plot showing the estimated prevalence of *Pfmdr1* drug resistance genes at codon Y184F in *Plasmodium falciparum* in Ethiopia from 2007–2014 and 2015–2025: A systematic review and meta-analysis. 2025

Subgroup analyses were carried out in this meta-analysis according to the study year. According to research conducted between 2007 and 2014, the prevalence of *Pfmdr1* drug resistance genes at codon Y184F was 66.0% (95% CI: 31–101). However, its incidence rose to 91.0% (95% CI: 83–100) between 2015 and 2025 (Fig. 4).

### Pooled prevalence of *pfmdr1* D1246Y mutations

**Fig. 5:**
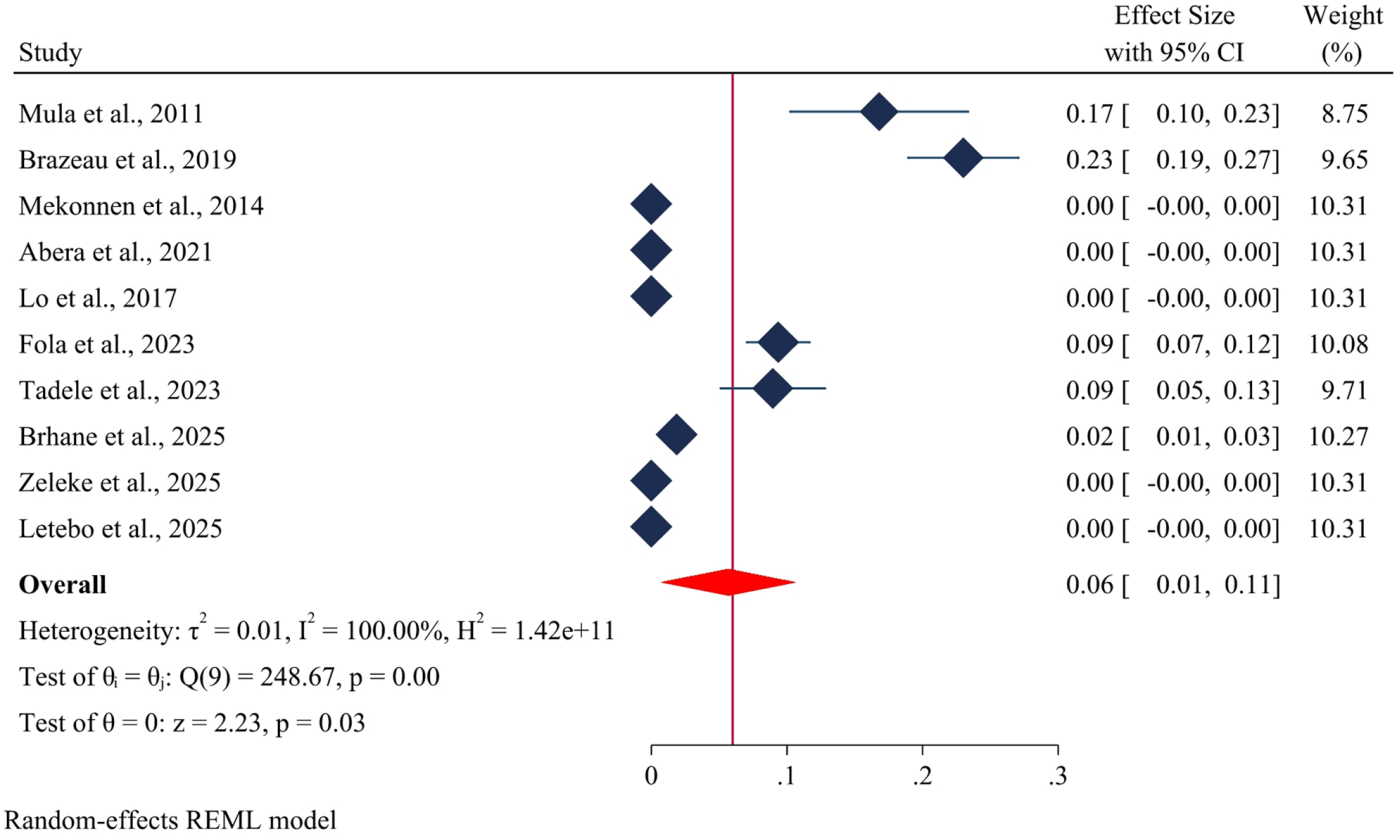
Forest plot depicting the prevalence of *Pfmdr1* drug resistance genes at codon D1246Y in *Plasmodium falciparum* in Ethiopia: A systematic review and meta-analysis. 2025

The overall estimated pooled prevalence of drug-resistant *Plasmodium falciparum Pfmdr1* genes at codon D1246Y was 6% (95% CI 1–11) (Fig. 5).

### Pooled prevalence of *kelch13* (R622I) mutations

**Fig. 6:**
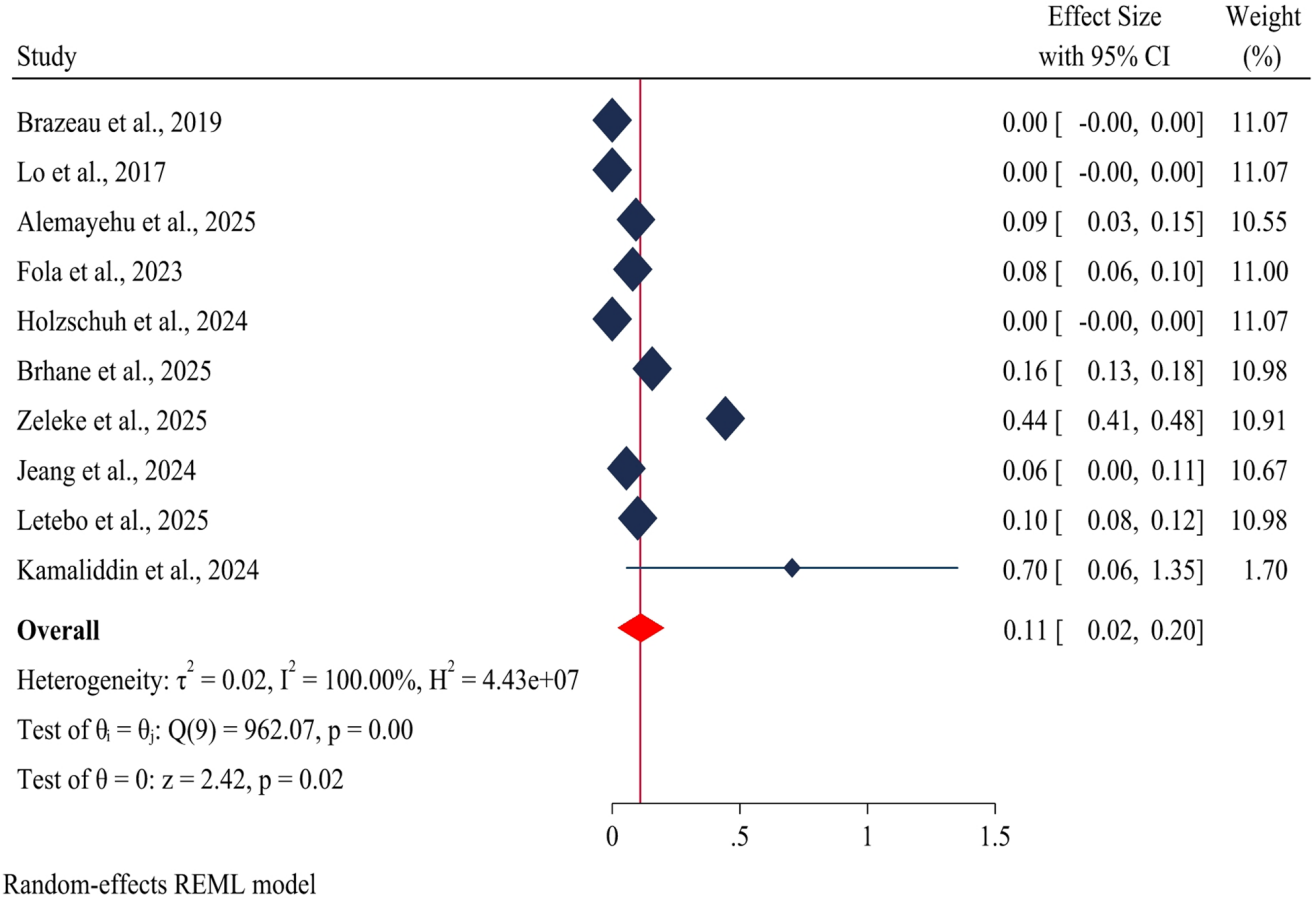
Forest plot depicting the estimated prevalence of *kelch13* (R622I) drug resistance genes in *Plasmodium falciparum* in Ethiopia: A systematic review and meta-analysis. 2025

The estimated pooled prevalence of the *kelch13* R622I mutation in Ethiopia was 11% (95%CI: 2–20%) (Fig. 6).

### Pooled prevalence of *kelch13* (P441L) mutations

**Fig. 7:**
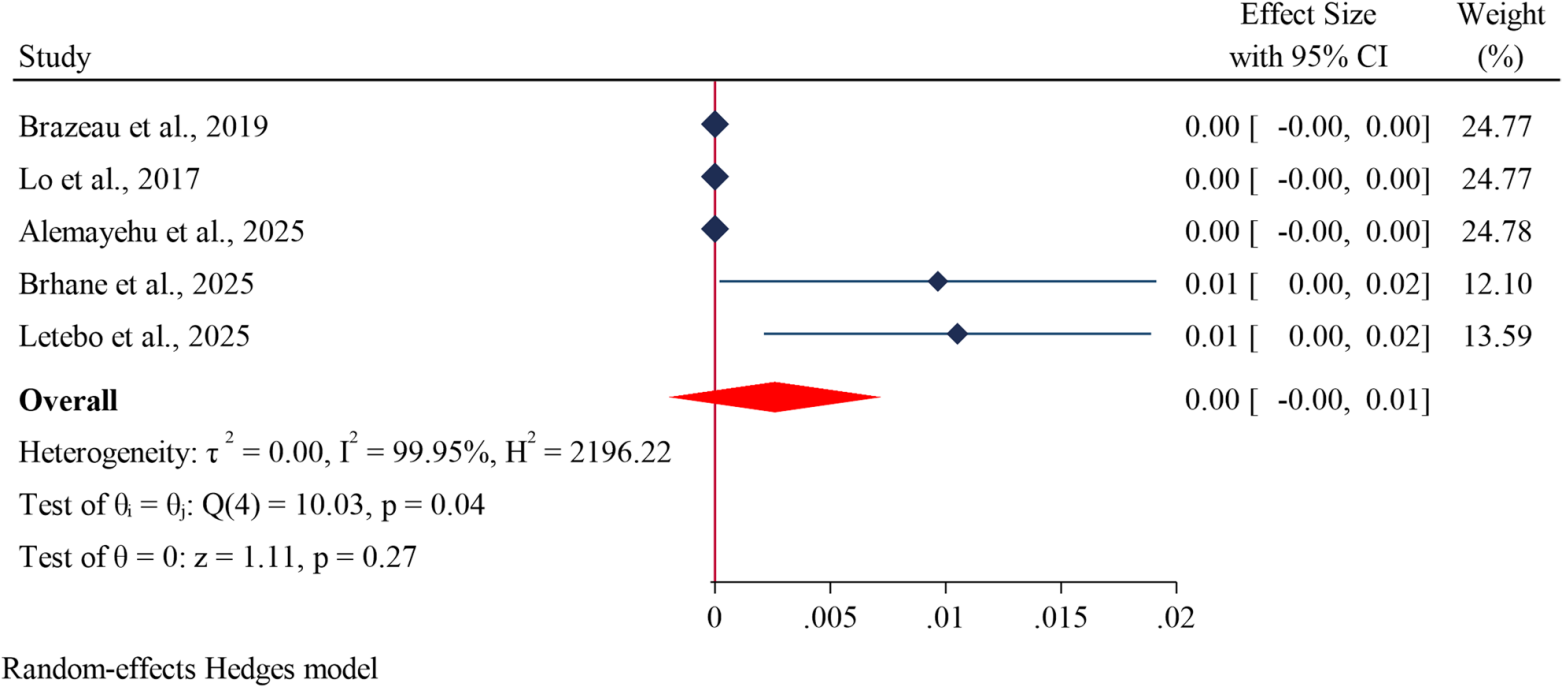
Forest plot showing the estimated prevalence of *kelch13* drug resistance genes at P441L codon in *Plasmodium falciparum* in Ethiopia: A systematic review and meta-analysis. 2025

The estimated pooled prevalence of the kelch13 P441L mutation was 0% (Fig. 7). This indicates that the P441L mutation is currently not detectable or is extremely rare in the populations among studies included in this meta-analysis.

### Pooled prevalence of *kelch13* (A675V) mutations

**Fig. 8:**
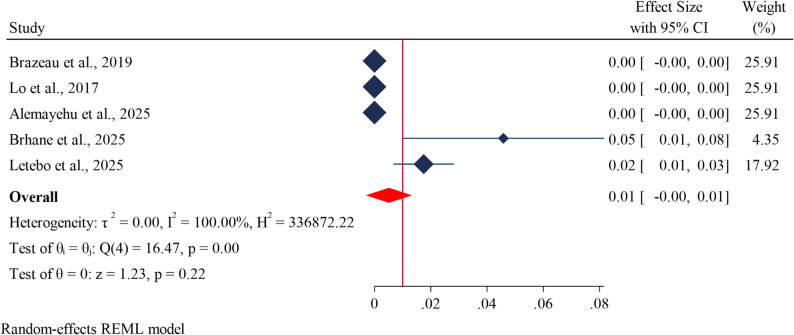
Forest plot showing the estimated prevalence of *pfk13* drug resistance genes at A675V codon in *Plasmodium falciparum* in Ethiopia: A systematic review and meta-analysis. 2025

The estimated pooled prevalence of the *kelch13* mutation at A675V codon was 1% (95% CI: 0.00–0.1%) (Fig. 8).

## Discussion

In Ethiopia, malaria transmission varies significantly across different ecological zones, from highlands to lowlands. Each region is home to distinct species of Anopheles mosquitoes, which possess varying abilities to transmit the malaria parasite. Areas with consistently high transmission rates, such as Gambella and Benishangul-Gumuz, are likely to experience increased pressure for the development of resistance mutations due to a larger overall parasite population and more frequent use of antimalarial medications. In contrast, regions with low transmission rates may see a slower emergence of resistance, as there are fewer opportunities for new mutations to develop and be selected [60].

This systematic review and meta-analysis determined the pooled prevalence of the *kelch13* and *Pfmdr1* antimalaria drug resistance gene mutation in Ethiopia via data from fifteen reputable scientific studies. Antimalarial drug distribution and usage patterns vary significantly across different regions. Since 2004, Ethiopia has relied on artemether-lumefantrine (AL) as the primary treatment for uncomplicated *Plasmodium falciparum* malaria. The analysis of the *Pfmdr1* Y184F mutation revealed an overall prevalence of 77%, which aligns with its known role in decreasing susceptibility to lumefantrine. These high mutation frequencies likely indicate strong selective pressure from AL and high adherence to treatment in certain areas. In contrast, regions where chloroquine or amodiaquine have been more commonly used, either historically or currently, often exhibit distinct resistance profiles.

This finding was higher than that reported in Nepal (44%) [61], Senegal (67.6%) [62], East Africa (58.3%) [63], India (54%) [64], China (60.7%, 72.2%) [65, 66], and Nigeria (29.27) [67]. This may be attributed to similar transmission intensities and malaria endemics, comparable interventions and control measures, and analogous migration and population movements [68].

In contrast, this finding was lower than those reported in Sudan (88.1%) [69], India (99.16%) [70], and Yemen (100%) [71]. The geographical heterogeneity in drug resistance is influenced by regional disparities in the deployment of antimalarial treatments, including both recommended regimens and practices of overuse. Additionally, the historical use of drugs such as chloroquine and the inherent genetic diversity within *Plasmodium falciparum* populations play significant roles. Collectively, these factors contribute to the distinct spatial distributions of critical resistance alleles, such as *Pfmdr1* Y184F. Additionally, differing prevalence rates between countries may result from variations in the timing of public health policy adjustments and the continued use of chloroquine for chemoprophylaxis in children with sickle cell disease [63, 72]. The prevalence of the *Pfmdr1* Y184F mutation has significantly increased over time, as indicated by a subgroup analysis by year. It rose sharply from 62.0% in studies conducted between 2007 and 2014 to 91.0% in studies from 2015 to 2025. This rapid increase in the *Pfmdr1* Y184F mutation prevalence in Ethiopia presents a serious challenge to current malaria treatment protocols, highlighting the urgent need for policymakers to implement new strategies and identify alternative drug regimens for effective malaria control [73].

The estimated pooled prevalence of *Plasmodium falciparum* with the *Pfmdr1* D1246Y mutation was 6.0%. This figure is lower than the 48.5% reported in a study conducted in Nigeria [74]. However, it is higher than findings from several other studies: 3.66% in Nigeria [67], 12% in Rwanda [75], 2.1% in Nigeria [76], 1.8% in Sierra Leone [77], and 3.91% in Cameroon [78]. The significant differences in the reported prevalence of *Pfmdr1* D1246Y can largely be attributed to high geographical variation within populations, as certain antimalarial agents, such as lumefantrine, may be selectively favored. Additionally, the timing of each study and the population participated, such as testing only symptomatic patients can significantly influence the observed dynamics of the disease [79].

The emergence of Artemisinin Resistance (ART-R), first reported in Southeast Asia in 2008, has more recently been confirmed in several African countries, including Ethiopia [23, 57], Sudan [80], Tanzania [81], and Kenya [82]. ART-R is phenotypically expressed as delayed parasite clearance and is primarily linked to mutations in the *Plasmodium falciparum kelch13* (*Pfkelch13* or *PfK13*) gene’s β-propeller domain [81, 83]. Consequently, this mutation of Plasmodium falciparum kelch13 is believed to contribute to slower parasite clearance after ACT, the primary clinical sign of partial ART-R [84]. To date, the WHO has validated a list of mutations causally associated with ART-R, which typically includes 13 non-synonymous mutations thus are F446I, N458Y, C469Y, M476I, Y493H, R539T, I543T, P553L, R561H, P574L, C580Y, R622I, and A675V [85]. Furthermore, nine other mutations are categorized as candidate or associated markers, including P441L, G449A, C469F, A481V, R515K, P527H, N537I/D, G538V, and V568G [86]. Fhe446Ile, Met476Ile, Pro553Leu, Val568Gly, Pro574Leu, and Ala675Val demonstrated a strong correlation with delayed parasite clearance in patients (in vivo) [87, 88]. However, the WHO has only formally validated three of these six as confirmed ART-R markers, requiring proof of a reduced response to Artemisinin in laboratory (in vitro) settings. These validated markers are Phe446Ile, Met476Ile, and Pro553Leu [86, 89].

The meta-analysis estimated that the pooled incidence of the *Plasmodium falciparum PfKelch13* R622I mutation in Ethiopia was 11%. This prevalence level is a significant finding, as it exceeds the established threshold for concern, indicating the documented emergence and development of resistance to Artemisinin a critical component of first-line malaria treatment within the region. This finding aligns with a report from neighboring Eritrea, which documented a prevalence of 11.9% [90]. However, our estimate is lower than those reported in several other African nations, including South Sudan (22.0%) [80], Namibia (33.2%) [91], Uganda (38%) [92] and Zambia (51%) [93]. Conversely, the estimated prevalence in Ethiopia is higher than the rates documented in several East African countries. Notably lower prevalence were reported in northwest Tanzania (0.03%) [39], Kenya (5.1%) [94], Tanzania (0.03%) [39], and Kenya (0.15%) [95]. In Ethiopia, the landscape of malaria resistance is significantly influenced by high levels of population mobility. Expansive internal and cross-border corridors, driven by agricultural labor cycles, trade, and regional displacement, serve as conduits for the spread of resistant *P. falciparum* lineages.

Notably, the validated kelch13 R622I mutation has been identified in northwestern Ethiopian sites bordering Sudan and has also been documented in Eritrea and South Sudan. This shared genetic signature strongly indicates cross-border gene flow facilitated by regional connectivity. Such human-mediated dispersal not only synchronizes resistance profiles across the Horn of Africa but also requires a coordinated multinational approach to genomic surveillance and drug policy [57].

In this meta-analysis, the pooled prevalence of the PfKelch13-P441L mutation was 0%, indicating its absence or extreme rarity across the combined study regions. This result aligns with the low frequencies reported in Burundi (0%) [96] and Angola < 0.5% [97] suggesting that the mutation is not established in these regions. However, this finding sharply contrasts with the high prevalence rates observed in other parts of Africa, such as 69% in Uganda [98] and 20% in Tanzania [98]. An intermediate, low-prevalence pattern is observed in West Africa (1.0%) [99], Zambia (1.39%) [100] and the Democratic Republic of the Congo (1.67%) [101]. This clear geographical gradient is the most salient finding of our study.

This meta-analysis quantifies the pooled prevalence of the *Plasmodium falciparum PfKelch13*-A675V mutation, a crucial marker for monitoring Artemisinin partial resistance. The overall prevalence of *PfKelch13*-A675V was found to be 1%, establishing a baseline while indicating significant regional heterogeneity that necessitates careful interpretation. Although this is lower than the estimate for the R622I mutation, the presence of the A675V mutation warrants continued surveillance because of its potential role as a marker for emerging resistance. This pooled result is comparable to findings from Namibia (1.2%) [91], but lower than the rates reported in Kenya (2.9%) [95] and Rwanda 6.4% [102].

This systematic review and meta-analysis provide valuable insights for malaria control by investigating the prevalence and temporal trends of key antimalarial drug resistance gene mutations in Ethiopia. It highlights the alarmingly high and increasing prevalence of the *Pfmdr1* Y184F mutation, which has reached 91% in recent years. This mutation is associated with reduced efficacy of lumefantrine, a component of the first-line ACT. The evidence underscores the urgent need to review and potentially revise national treatment guidelines, consider alternative ACT formulations, or implement region-specific drug policies. Additionally, the study establishes baseline prevalence for emerging *kelch13* mutations linked to Artemisinin partial resistance, facilitating early detection and targeted surveillance. By consolidating fragmented data into a national estimate, this review supports evidence-based decision-making, prioritizes molecular surveillance in high-risk regions, and informs regional strategies for containing the spread of resistance. Ultimately, it aids in sustaining the effectiveness of current treatments and guiding future intervention strategies in Ethiopia and beyond.

### Strengths and Limitations

Key strengths of this systematic review and meta-analysis include adherence to a standardized protocol and the use of established international tools for critically assessing the quality of the included research. The clear identification and reported pooled prevalence of the Pfmdr1 marker are also positive aspects. Conversely, a potential limitation is that the prevalence estimates for the *kelch13* gene (R622I, P441L, and A675V) and *Pfmdr1* (Y184F and D1246Y) drug resistance genes may be skewed due to the exclusive inclusion of English-language publications. Additionally, the cross-sectional, laboratory-based nature of the original studies may mean that the outcome variable was affected by extraneous confounding variables.

## Conclusion

This systematic review and meta-analysis highlights the changing landscape of *Plasmodium falciparum* drug resistance, revealing distinct and actionable epidemiological patterns. Although confirmed markers of Artemisinin partial resistance in the *kelch13* gene remain relatively rare, the significant rise in the frequency of the *Pfmdr1* Y184F mutation is a critical finding. Its dramatic increase from 62% to 91% over the past two decades indicates a substantial shift in the parasite population, driven by ongoing drug pressure. This mutation affects susceptibility to essential partner drugs, such as lumefantrine and amodiaquine, posing a direct threat to the effectiveness of widely used artemisinin-based combination therapies (ACTs).

These findings have major implications for global malaria control. The high and increasing prevalence of *Pfmdr1* Y184F compromises the long-term effectiveness of current first-line treatment regimens and necessitates a proactive, evidence-based revision of treatment policies. Therefore, continuous, high-resolution molecular surveillance must be established as a key component of national malaria control programs. Surveillance data should be integrated in real time to guide regional drug policy decisions, including the rotation or substitution of partner drugs within ACTs. Additionally, this evidence emphasizes the urgent need to expedite the development and implementation of novel non-artemisinin-based combinations to ensure effective treatment options remain available. In summary, strategic management of antimalarial drug resistance, supported by vigilant molecular monitoring and proactive policy adjustments, is crucial to preserving recent advancements and achieving sustainable malaria elimination.

## Data Availability

All data supporting the findings of this study are included in this published article.

## Abbreviations

ACT: Artemisinin-based combination therapy
ARTs: Artemisinin derivatives
CQ: Chloroquine
HRM: High Resolution Melting
MIP: Molecular inversion probes
PCR-RFLP: Polymerase Chain Reaction - Restriction Fragment Length Polymorphism
*Pfatpase*: *Plasmodium falciparum* ATPase gene
*Pfdhfr*: *Plasmodium falciparum* dihydrofolate reductase gene
*Pfdhps*: *Plasmodium falciparum* dihydropteroate synthase gen
*Pfk13*: *Plasmodium falciparum kelch* 13 gene
*Pfmdr1*: *Plasmodium falciparum* multidrug resistance 1 gene
SNNP: South nation nationality and people
SP: Sulfadoxine-pyrimethamine
WHO: World Health Organization

## References

[1] Monroe A, Williams NA, Ogoma S, Karema C, Okumu F. Reflections on the 2021 World Malaria Report and the future of malaria control. Malar J. 2022;21(1):154. 35624483 10.1186/s12936-022-04178-7 PMC9137259

[2] Oladipo HJ, Tajudeen YA, Oladunjoye IO, Yusuff SI, Yusuf RO, Oluwaseyi EM, AbdulBasit MO, Adebisi YA, El-Sherbini MS. Increasing challenges of malaria control in sub-Saharan Africa: Priorities for public health research and policymakers. Annals Med Surg. 2022;81:104366. 10.1016/j.amsu.2022.104366 PMC9421173 36046715

[3] Djihinto OY, Medjigbodo AA, Gangbadja AR, Saizonou HM, Lagnika HO, Nanmede D, Djossou L, Bohounton R, Sovegnon PM, Fanou M-J. Malaria-transmitting vectors microbiota: Overview and interactions with anopheles mosquito biology. Front Microbiol. 2022;13:891573. 35668761 10.3389/fmicb.2022.891573 PMC9164165

[4] Snounou G, Sharp PM, Culleton R. The two parasite species formerly known as Plasmodium ovale. Trends Parasitol. 2024;40(1):21–7. 38040603 10.1016/j.pt.2023.11.004

[5] Venkatesan P. WHO world malaria report 2024. Lancet Microbe. 2025;6(4). 10.1016/j.lanmic.2025.101073 39923782

[6] Ababa A. Diagnosis and treatment guidelines for health workers in Ethiopia. 2004. Addis Ababa.

[7] Barman K, Goswami P. Recent Advances in Diagnostics and Therapeutic Interventions for Drug-Resistant Malaria. ACS Infect Dis. 2025;11(6):1296–332. 40326084 10.1021/acsinfecdis.4c00962

[8] Agbedahunsi JM, Cyril-Olutayo MC, Fadare RY, Ogundolie FA, Salaria D, Rolta R, Idowu SA, Oladele AB, Eso PD, Olatunji EO. Computational study of phytochemicals from Khaya grandifoliola (WELW) as potential inhibitors of the Plasmodium falciparum transketolase and putative antimalarial agents. Silico Pharmacol. 2025;13(2):87. 10.1007/s40203-025-00378-6 PMC12162438 40520960

[9] Cruz-Hurtado M, López-González MdL, Mondragón V. Sierra-Santoyo, *In vitro phase I metabolism of vinclozolin by human liver microsomes*. Xenobiotica. 2019;49(8):895–904. 30215542 10.1080/00498254.2018.1523485

[10] Prost R, Płaziński W. Natural Polymorphic Variants in the CYP450 Superfamily: A Review of Potential Structural Mechanisms and Functional Consequences. Int J Mol Sci. 2025;26(16):7797. 40869119 10.3390/ijms26167797 PMC12386198

[11] Charoenchokthavee W, Areepium N, Panomvana D, Sriuranpong V. Effects of CYP2D6 and CYP3A5 polymorphisms on tamoxifen and its metabolites in Thai breast cancer patients. Breast Cancer Targets Therapy. 2017:249–56. 10.2147/BCTT.S125745 PMC5399972 28450788

[12] Latham BD, Montazeri P, Lobo LF, Fallon JK, Jackson KD. Impact of variation in CYP3A and CYP2C8 on tucatinib metabolic clearance in human liver microsomes. Drug Metab Dispos. 2025;53(5):100061. 40233610 10.1016/j.dmd.2025.100061 PMC13095627

[13] de Pereira LW, Fuzii HT, Villanova FE, Nunes Cardoso Mello AG, de Sena MPM, Dias Ferreira MV, Fernandes JL, Vieira. Influence of CYP2C8 Polymorphism on the Exposure to Chloroquine in Patients with Malaria by Plasmodium vivax—A Preliminary Study. Int J Environ Res Public Health. 2025;22(3):336. 40238288 10.3390/ijerph22030336 PMC11941787

[14] Sanyaolu A, Marinkovic A, Prakash S, Balendra V, Shazley O, Gardellini T, Jan A, Younis K, Okorie C, Izurieta R. Emerging Molecular Mechanisms in Malaria Pathogenesis and Novel Therapeutic Approaches: A Focus on P. falciparum Malaria. Biomolecules. 2025;15(7):1038. 40723909 10.3390/biom15071038 PMC12293666

[15] Tran KT, Nguyen TD, Weissman DB, Li EZ, Mok S, Small-Saunders JL, Bousema T, Zupko RJ, Tran TN-A, Boni MF. Effects of recombination on multi-drug resistance evolution in Plasmodium falciparum malaria. PLoS Comput Biol. 2025;21(8):e1013401. 40853961 10.1371/journal.pcbi.1013401 PMC12377563

[16] Nikiema S, Soulama I, Ampofo GD, Nikiema M, Zouré AA, Sombié S, Sawadogo S, Ouedraogo N, Sermé SS, Sawadogo H. Influence of genetic factors of humans, mosquitoes and parasites, on the evolution of Plasmodium falciparum infections, malaria transmission and genetic control methods: a review of the literature. BMC Med Genom. 2025;18(1):100. 10.1186/s12920-025-02165-w PMC12131405 40457392

[17] Igwe MC, Ogbuabor OA, Obeagu EI. Evolutionary biology of antimalarial drug resistance: Understanding of the evolutionary dynamics. Medicine. 2025;104(11):e41878. 40101051 10.1097/MD.0000000000041878 PMC11922465

[18] Thomford NE, Kellermann T, Twum JA, Anyimadu J, Dixon C, Sappor D, Blackhurst D, Barnie PA, Ryabinina O, Nyarko SB. Drug-drug interaction between dolutegravir and artemether-lumefantrine in HIV and malaria mono-and co-infections: a pharmacogenetic analysis from Ghana. AIDS Res Therapy. 2025;22(1):1–18. 10.1186/s12981-025-00787-9 PMC12398046 40886013

[19] Cheng W, Song X, Tan H, Wu K, Li J. Molecular surveillance of anti-malarial resistance pfcrt, pfmdr1, and pfk13 polymorphisms in African Plasmodium falciparum imported parasites to Wuhan, China. Malar J. 2021;20(1):209. 33933099 10.1186/s12936-021-03737-8 PMC8087876

[20] Lekostaj JK, Amoah LE, Roepe PD. A single S1034C mutation confers altered drug sensitivity to PfMDR1 ATPase activity that is characteristic of the 7G8 isoform. Mol Biochem Parasitol. 2008;157(1):107–11. 18006157 10.1016/j.molbiopara.2007.09.008 PMC2211713

[21] Xie SC, Ralph SA, Tilley L. K13, the cytostome, and artemisinin resistance. Trends Parasitol. 2020;36(6):533–44. 32359872 10.1016/j.pt.2020.03.006

[22] Kong X, Feng J, Xu Y, Yan G, Zhou S. Molecular surveillance of artemisinin resistance-related Pfk13 and pfcrt polymorphisms in imported Plasmodium falciparum isolates reported in eastern China from 2015 to 2019. Malar J. 2022;21(1):369. 36464686 10.1186/s12936-022-04398-x PMC9719650

[23] Brhane BG, Fola AA, Nigussie H, Leonetti A, Kassa M, Hailgiorgis H, Wuletaw Y, Abera A, Mohammed H, Sime H. Rising prevalence of Plasmodium falciparum Artemisinin partial resistance mutations in Ethiopia. Commun Med. 2025;5(1):297. 40681807 10.1038/s43856-025-01008-0 PMC12274420

[24] Mihreteab S, Platon L, Berhane A, Stokes BH, Warsame M, Campagne P, Criscuolo A, Ma L, Petiot N. Doderer-Lang, *Increasing prevalence of artemisinin-resistant HRP2-negative malaria in Eritrea*. N Engl J Med. 2023;389(13):1191–202. 37754284 10.1056/NEJMoa2210956 PMC10539021

[25] Uwimana A, Umulisa N, Venkatesan M, Svigel SS, Zhou Z, Munyaneza T, Habimana RM, Rucogoza A, Moriarty LF, Sandford R. Association of Plasmodium falciparum kelch13 R561H genotypes with delayed parasite clearance in Rwanda: an open-label, single-arm, multicentre, therapeutic efficacy study. Lancet Infect Dis. 2021;21(8):1120–8. 33864801 10.1016/S1473-3099(21)00142-0 PMC10202849

[26] Avcı KD, Karakuş M, Kart K, Yaşar. Molecular survey of pfmdr-1, pfcrt, and pfk13 gene mutations among patients returning from Plasmodium falciparum endemic areas to Turkey. Malar J. 2024;23(1):286. 39334180 10.1186/s12936-024-05107-6 PMC11437951

[27] Laird VR, Plucinski MM, Venkatesan M, Rondini KA, Randrianarivelojosia M, Andriamananjara MN, Moonga H, Ishengoma DS, Chidimatembue A, Dimbu PR. Plasmodium falciparum multidrug resistance 1 gene polymorphisms associated with outcomes after anti-malarial treatment. Malar J. 2025;24(1):186. 40506728 10.1186/s12936-025-05248-2 PMC12160118

[28] Veiga MI, Dhingra SK, Henrich PP, Straimer J, Gnädig N, Uhlemann A-C, Martin RE, Lehane AM, Fidock DA. Globally prevalent PfMDR1 mutations modulate Plasmodium falciparum susceptibility to artemisinin-based combination therapies. Nat Commun. 2016;7(1):11553. 27189525 10.1038/ncomms11553 PMC4873939

[29] Mula P, Fernández-Martínez A, de Lucio A, Ramos JM, Reyes F, González V, Benito A, Berzosa P. Detection of high levels of mutations involved in anti-malarial drug resistance in Plasmodium falciparum and Plasmodium vivax at a rural hospital in southern Ethiopia. Malar J. 2011;10(1):214. 21810256 10.1186/1475-2875-10-214 PMC3161020

[30] Tadele G, Jawara A, Oboh M, Oriero E, Dugassa S, Amambua-Ngwa A, Golassa L. Clinical isolates of uncomplicated falciparum malaria from high and low malaria transmission areas show distinct pfcrt and pfmdr1 polymorphisms in western Ethiopia. Malar J. 2023;22(1):171. 37270589 10.1186/s12936-023-04602-6 PMC10239113

[31] Srimuang K, Miotto O, Lim P, Fairhurst RM, Kwiatkowski DP, Woodrow CJ, Imwong M. .t.A. Collaboration, *Analysis of anti-malarial resistance markers in pfmdr1 and pfcrt across Southeast Asia in the Tracking Resistance to Artemisinin Collaboration*. Malar J. 2016;15:1–12. 27825353 10.1186/s12936-016-1598-6 PMC5101715

[32] Dondorp AM, Nosten F, Yi P, Das D, Phyo AP, Tarning J, Lwin KM, Ariey F, Hanpithakpong W, Lee SJ. Artemisinin resistance in Plasmodium falciparum malaria. N Engl J Med. 2009;361(5):455–67. 19641202 10.1056/NEJMoa0808859 PMC3495232

[33] Pacheco C, Moreno J, Herrera F. A high number of pfmdr1 gene copies in P. falciparum from Venezuela. Parasitol Res. 2019;118(10):3085–9. 31396714 10.1007/s00436-019-06409-4

[34] Win AA, Imwong M, Kyaw MP, Woodrow CJ, Chotivanich K, Hanboonkunupakarn B, Pukrittayakamee S. K13 mutations and pfmdr1 copy number variation in Plasmodium falciparum malaria in Myanmar. Malar J. 2016;15:1–7. 26911145 10.1186/s12936-016-1147-3 PMC4765153

[35] Labadie-Bracho M, Adhin MR. Increased pfmdr1 copy number in Plasmodium falciparum isolates from Suriname. Tropical Med Int Health. 2013;18(7):796–9. 10.1111/tmi.12118 23621761

[36] Tajebe A, Aemero M, Francis K, Magoma G. Identification of chloroquine resistance Pfcrt-K76T and determination of Pfmdr1-N86Y copy number by SYBR Green I qPCR. Asian Pac J Trop Biomed. 2015;5(3):208–20.

[37] Aydemir O, Janko M, Hathaway NJ, Verity R, Mwandagalirwa MK, Tshefu AK, Tessema SK, Marsh PW, Tran A, Reimonn T. Drug-resistance and population structure of Plasmodium falciparum across the Democratic Republic of Congo using high-throughput molecular inversion probes. J Infect Dis. 2018;218(6):946–55. 29718283 10.1093/infdis/jiy223 PMC6093412

[38] Conrad MD, Asua V, Garg S, Giesbrecht D, Niaré K, Smith S, Namuganga JF, Katairo T, Legac J, Crudale RM. Evolution of partial resistance to artemisinins in malaria parasites in Uganda. N Engl J Med. 2023;389(8):722–32. 37611122 10.1056/NEJMoa2211803 PMC10513755

[39] Juliano JJ, Giesbrecht DJ, Simkin A, Fola AA, Lyimo BM, Pereus D, Bakari C, Madebe RA, Seth MD, Mandara CI. Country wide surveillance reveals prevalent artemisinin partial resistance mutations with evidence for multiple origins and expansion of high level sulfadoxine-pyrimethamine resistance mutations in northwest Tanzania. MedRxiv. 2023.

[40] Cantsilieris S, Stessman HA, Shendure J, Eichler EE. Targeted capture and high-throughput sequencing using molecular inversion probes (MIPs). Genotyping: Methods and Protocols. Springer; 2016. pp. 95–106. 10.1007/978-1-4939-6442-0_6 PMC5484527 27822858

[41] Neafsey DE, Taylor AR, MacInnis BL. Advances and opportunities in malaria population genomics. Nat Rev Genet. 2021;22(8):502–17. 33833443 10.1038/s41576-021-00349-5 PMC8028584

[42] Authority EP. National report of Ethiopia. in The United Nations Conference on Sustainable Development (Rio 20+). 2012.

[43] Dufera M, Kenea O, Tadele G. Malaria incidence and associated risk factors in and around Anger Gute Town, Western Ethiopia. 2020.

[44] Grossman P, Niemann L, Schmidt S, Walach H. Mindfulness-based stress reduction and health benefits: A meta-analysis. J Psychosom Res. 2004;57(1):35–43. 15256293 10.1016/S0022-3999(03)00573-7

[45] Tamiru A, Tolossa T, Regasa B, Mosisa G. Prevalence of asymptomatic malaria and associated factors in Ethiopia: systematic review and meta-analysis. SAGE Open Med. 2022;10:20503121221088085. 35433001 10.1177/20503121221088085 PMC9006361

[46] Munn Z, Moola S, Lisy K, Riitano D, Tufanaru C, Aromataris E. *Systematic reviews of prevalence and incidence*, in *JBI manual for evidence synthesis*. Australia: JBI Adelaide; 2020. pp. 117–217.

[47] Moher D, Liberati A, Tetzlaff J, Altman DG and t. PRISMA Group*. Preferred reporting items for systematic reviews and meta-analyses: the PRISMA statement. Ann Intern Med. 2009;151(4):264–9. 19622511 10.7326/0003-4819-151-4-200908180-00135

[48] Brazeau NF, Assefa A, Mohammed H, Seme H, Tsadik AG, Parr JB, Keeler C, Hathaway NJ, Meshnick SR, Bailey JA. Pooled deep sequencing of drug resistance loci from Plasmodium falciparum parasites across Ethiopia. Am J Trop Med Hyg. 2019;101(5):1139. 31516103 10.4269/ajtmh.19-0142 PMC6838589

[49] Mekonnen SK, Aseffa A, Berhe N, Teklehaymanot T, Clouse RM, Gebru T, Medhin G, Velavan TP. Return of chloroquine-sensitive Plasmodium falciparum parasites and emergence of chloroquine-resistant Plasmodium vivax in Ethiopia. Malar J. 2014;13(1):244. 24964730 10.1186/1475-2875-13-244 PMC4230645

[50] Heuchert A, Abduselam N, Zeynudin A, Eshetu T, Löscher T, Wieser A, Pritsch M, Berens-Riha N. Molecular markers of anti-malarial drug resistance in southwest Ethiopia over time: regional surveillance from 2006 to 2013. Malar J. 2015;14(1):208. 25986047 10.1186/s12936-015-0723-2 PMC4490604

[51] Abera D, Kibet CK, Degefa T, Amenga-Etego L, Bargul JL, Golassa L. Genomic Anal reveals Indep Evol Plasmodium falciparum populations Ethiopia Malar J. 2021;20(1):129. 10.1186/s12936-021-03660-y PMC7934276 33663492

[52] Lo E, Hemming-Schroeder E, Yewhalaw D, Nguyen J, Kebede E, Zemene E, Getachew S, Tushune K, Zhong D, Zhou G. Transmission dynamics of co-endemic Plasmodium vivax and P. falciparum in Ethiopia and prevalence of antimalarial resistant genotypes. PLoS Negl Trop Dis. 2017;11(7):e0005806. 28746333 10.1371/journal.pntd.0005806 PMC5546713

[53] Alemayehu AA, Castañeda Mogollón D, Belay SG, Tesfa H, Mohon AN, Balasingam N, Bayih AG, Ashraf S, Pillai DR. Expansion of the *Plasmodium falciparum**Kelch*13 R622I mutation in Northwest Ethiopia. Open Forum Infectious Diseases. Oxford University Press US. 2025. 10.1093/ofid/ofaf279 PMC12130971 40463829

[54] Fola AA, Feleke SM, Mohammed H, Brhane BG, Hennelly CM, Assefa A, Crudal RM, Reichert E, Juliano JJ, Cunningham J. Plasmodium falciparum resistant to artemisinin and diagnostics have emerged in Ethiopia. Nat Microbiol. 2023;8(10):1911–9. 37640962 10.1038/s41564-023-01461-4 PMC10522486

[55] Holzschuh A, Ewnetu Y, Carlier L, Lerch A, Gerlovina I, Baker SC, Yewhalaw D, Haileselassie W, Berhane N, Lemma W. Plasmodium falciparum transmission highlands Ethiopia is driven closely Relat clonal parasites Mol Ecol. 2024;33(6):e17292. 10.1111/mec.17292 38339833

[56] Letebo A, Vanheer LN, Engdaw M, Alemayehu DH, Deressa JD, Adnew B, Ayele A, Keffale M, Demisse M, Seyoum T. Convergent Evolution of Artemisinin and Chloroquine Resistance in Ethiopian Plasmodium falciparum Parasites. VeriXiv. 2025;2(162):162.

[57] Jeang B, Zhong D, Lee M-C, Atieli H, Yewhalaw D, Yan G. Molecular surveillance of Kelch 13 polymorphisms in Plasmodium falciparum isolates from Kenya and Ethiopia. Malar J. 2024;23(1):36. 38287365 10.1186/s12936-023-04812-y PMC10823687

[58] Zeleke AJ, Fola AA, Tollefson GA, Niaré K, Leonetti A, Taropawala O, Marglous J, Crudale R, Brhane BG, Assefa A. Artemisinin resistant *kelch13 R622I and RDT *negativity approaching predominance in northern Ethiopia and emerging* C580Y *of African origin threaten falciparum malaria control*.* medRxiv, 2025: p. 2025.06. 23.25330019.

[59] Kamaliddin C, Burke-Gaffney J, Ashraf S, Castañeda-Mogollón D, Adamu A, Mekonen Tefa B, Wijesinghe A, Pussegoda E, Feleke SM, Pillai DR. A Countrywide Survey of* hrp2/3 *Deletions and* kelch13 *mutations Co-occurrence* in Ethiopia.* The J Infect Dis. 2024. 230(6):e1394-e1401. 10.1093/infdis/jiae373 PMC11646589 39083679

[60] Nguyen TD, Tran TN-A, Parker DM, White NJ, Boni MF. Antimalarial mass drug administration in large populations and the evolution of drug resistance. PLOS global public health. 2023;3(7):e0002200. 37494337 10.1371/journal.pgph.0002200 PMC10370688

[61] Ranjitkar S, Schousboe ML, Thomsen TT, Adhikari M, Kapel CM, Bygbjerg IC, Alifrangis M. Prevalence of molecular markers of anti-malarial drug resistance in Plasmodium vivax and Plasmodium falciparum in two districts of Nepal. Malar J. 2011;10(1):75. 21457533 10.1186/1475-2875-10-75 PMC3080351

[62] Wurtz N, Fall B, Pascual A, Diawara S, Sow K, Baret E, Diatta B, Fall KB, Mbaye PS, Fall F. Prevalence Mol markers Plasmodium falciparum drug Resist Dakar Senegal Malar J. 2012;11(1):197. 10.1186/1475-2875-11-197 PMC3470961 22694921

[63] Abebe W, Mekuanint A, Asmare Z, Woldesenbet D, Mihret Y, Setegn A, Emagneneh T. Prevalence of molecular markers of chloroquine resistance in malaria parasites in East Africa: A systematic review and meta-analysis. J Global Antimicrob Resist. 2025;41:117–37. 10.1016/j.jgar.2024.12.019 39725320

[64] Ozarkar A, Kanyal A, Dass S, Deshpande P, Deobagkar D, Karmodiya K. Analysis of drug resistance marker genes of Plasmodium falciparum after implementation of artemisinin-based combination therapy in Pune district, India. J Biosci. 2021;46(3):77. 34344849

[65] Xu C, Wei Q, Yin K, Sun H, Li J, Xiao T, Kong X, Wang Y, Zhao G, Zhu S. Surveillance of antimalarial resistance Pfcrt, Pfmdr1, and Pfkelch13 polymorphisms in African Plasmodium falciparum imported to Shandong Province, China. Sci Rep. 2018;8(1):12951. 30154519 10.1038/s41598-018-31207-w PMC6113250

[66] She D, Wang Z, Liang Q, Lu L, Huang Y, Zhang K, An D, Wu J. Polymorphisms of pfcrt, pfmdr1, and K13-propeller genes in imported falciparum malaria isolates from Africa in Guizhou province, China. BMC Infect Dis. 2020;20(1):513. 32677899 10.1186/s12879-020-05228-8 PMC7364468

[67] Ikegbunam MN, Nkonganyi CN, Thomas BN, Esimone CO, Velavan TP, Ojurongbe O. Analysis of Plasmodium falciparum Pfcrt and Pfmdr1 genes in parasite isolates from asymptomatic individuals in Southeast Nigeria 11 years after withdrawal of chloroquine. Malar J. 2019;18(1):343. 31590670 10.1186/s12936-019-2977-6 PMC6781387

[68] Ryan SJ, Lippi CA, Zermoglio F. Shifting transmission risk for malaria in Africa with climate change: a framework for planning and intervention. Malar J. 2020;19(1):170. 32357890 10.1186/s12936-020-03224-6 PMC7193356

[69] Hussien M, Abdel Hamid MM, Elamin EA, Hassan AO, Elaagip AH, Salama AHA, Abdelraheem MH, Mohamed AO. Antimalarial drug resistance molecular makers of Plasmodium falciparum isolates from Sudan during 2015–2017. PLoS ONE. 2020;15(8):pe0235401. 10.1371/journal.pone.0235401 PMC7446868 32817665

[70] Mittra P, Vinayak S, Chandawat H, Das MK, Singh N, Biswas S, Dev V, Kumar A, Ansari MA, Sharma YD. Progressive increase in point mutations associated with chloroquine resistance in Plasmodium falciparum isolates from India. J Infect Dis. 2006;193(9):1304–12. 16586369 10.1086/502979

[71] Atroosh WM, Al-Mekhlafi HM, Al-Jasari A, Sady H, Dawaki SS, Elyana FN, Al-Areeqi MA, Nasr NA, Abdulsalam AM, Subramaniam LR. Different patterns of pfcrt and pfmdr1 polymorphism in Plasmodium falciparum isolates from Tehama region, Yemen. PeerJ. 2016;4:e2191. 27478699 10.7717/peerj.2191 PMC4950566

[72] Mbogo GW, Nankoberanyi S, Tukwasibwe S, Baliraine FN, Nsobya SL, Conrad MD, Arinaitwe E, Kamya M, Tappero J, Staedke SG. Temporal changes in prevalence of molecular markers mediating antimalarial drug resistance in a high malaria transmission setting in Uganda. Am J Trop Med Hyg. 2014;91(1):54. 24799371 10.4269/ajtmh.13-0647 PMC4080569

[73] Menard D, Dondorp A. Antimalarial drug resistance: a threat to malaria elimination. Cold Spring Harbor Perspect Med. 2017;7(7):a025619. 10.1101/cshperspect.a025619 PMC5495053 28289248

[74] Patrick OJ, Amodu OK, Sokan-Adeaga AA, Sokan-Adeaga MA, Kotera Y. Prevalence and distribution of Plasmodium falciparum multidrug resistant 1 D1246Y allele among children in Ibadan Southwest, Nigeria. Sci Rep. 2025;15(1):9715. 40113916 10.1038/s41598-025-94668-w PMC11926228

[75] Zeile I, Gahutu J-B, Shyirambere C, Steininger C, Musemakweri A, Sebahungu F, Karema C, Harms G, Eggelte TA, Mockenhaupt FP. Molecular markers of Plasmodium falciparum drug resistance in southern highland Rwanda. Acta Trop. 2012;121(1):50–4. 21996622 10.1016/j.actatropica.2011.09.009

[76] Ajibaye S, Oboh-Imafidon M, Ajibola O, Olusola F, Egwu CO, Amambua-Ngwa B, Diop MF, Mohammed NI, D’Alessandro U, Falade CF. Molecular Surveillance of malaria in Nigeria reveals expansion of chloroquine-sensitive infections. medRxiv, 2025: p. 2025.07. 31.25332537. 10.1093/ofid/ofaf719 PMC12809531 41550699

[77] Chen H, Owusu-Kyei K, Fombah AE, Williams J, García-Fernández C, Rovira-Vallbona E, Bofill A, Quintó L, Figueroa-Romero A. Mac-Abdul, *Prevalence of Molecular Markers of Resistance to Antimalarial Drugs Three Years After Perennial Malaria Chemoprevention in Sierra Leone*. VeriXiv. 2025;2(114):114. 10.12688/gatesopenres.16367.1 PMC12508423 41080970

[78] Tuedom AGB, Sarah-Matio EM, Moukoko CEE, Feufack-Donfack BL, Maffo CN, Bayibeki AN, Awono-Ambene HP, Ayong L, Berry A, Abate L. Antimalarial drug resistance in the Central and Adamawa regions of Cameroon: Prevalence of mutations in P. falciparum crt, Pfmdr1, Pfdhfr and Pfdhps genes. PLoS ONE. 2021;16(8):e0256343. 34411157 10.1371/journal.pone.0256343 PMC8376100

[79] Dakorah MP, Aninagyei E, Attoh J, Adzakpah G, Tukwarlba I, Acheampong DO. Profiling antimalarial drug-resistant haplotypes in Pfcrt, Pfmdr 1, Pfdhps and Pfdhfr genes in Plasmodium falciparum causing malaria in the Central Region of Ghana: a multicentre cross-sectional study. Therapeutic Adv Infect Disease. 2025;12:20499361251319665. 10.1177/20499361251319665 PMC11833835 39968164

[80] L’Episcopia M, Talha AA, Nour BY, Sana IMA, Caspar E, Thiebaut L, Platon L, Chala B, Ma L, Golassa L. High Prevalence of Artemisinin-Resistant Plasmodium falciparum, Southeastern Sudan. Emerg Infect Dis. 2025;31(6):1211. 40439506 10.3201/eid3106.241810 PMC12123913

[81] Ishengoma DS, Mandara CI, Bakari C, Fola AA, Madebe RA, Seth MD, Francis F, Buguzi CC, Moshi R, Garimo I. Evidence of artemisinin partial resistance in northwestern Tanzania: clinical and molecular markers of resistance. Lancet Infect Dis. 2024;24(11):1225–33. 39159633 10.1016/S1473-3099(24)00362-1 PMC11511676

[82] Makau M, Kanoi BN, Mgawe C, Maina M, Abkallo H, Waweru H, Adung F, Gitaka J. Evidence of Partial Artemisinin Resistance in Malaria Endemic Lake Region, Busia County, Western, Kenya. No Title); 2024.

[83] Henrici RC, Sutherland CJ. Alternative pathway to reduced artemisinin susceptibility in Plasmodium falciparum. Proc Natl Acad Sci. 2018;115(50):12556–8. 30487214 10.1073/pnas.1818287115 PMC6294889

[84] Oyegbade SA, Mameh EO, Balogun DO, Aririguzoh V-GO, Akinduti PA. Emerging Plasmodium falciparum K13 gene mutation to artemisinin-based combination therapies and partner drugs among malaria-infected population in sub-Saharan Africa. Parasites Hosts Dis. 2025;63(2):109. 40452273 10.3347/PHD.24053 PMC12127821

[85] Organization WH. Report on antimalarial drug efficacy, resistance and response: 10 years of surveillance (2010–2019). World Health Organization. 2020.

[86] Organization WH. Artemisinin resistance and artemisinin-based combination therapy efficacy: status report. 2018.

[87] Bonnington CA, Phyo AP, Ashley EA, Imwong M, Sriprawat K, Parker DM, Proux S, White NJ, Nosten F. Plasmodium falciparum Kelch 13 mutations and treatment response in patients in Hpa-Pun District, northern Kayin State, Myanmar. Malar J. 2017;16(1):480. 29178921 10.1186/s12936-017-2128-x PMC5702082

[88] Wang J, Huang Y, Zhao Y, Ye R, Zhang D, Pan W. Introduction of F446I mutation in the K13 propeller gene leads to increased ring survival rates in Plasmodium falciparum isolates. Malar J. 2018;17(1):248. 29976207 10.1186/s12936-018-2396-0 PMC6034266

[89] Rosenthal PJ, Asua V, Bailey JA, Conrad MD, Ishengoma DS, Kamya MR, Rasmussen C, Tadesse FG, Uwimana A, Fidock DA. The emergence of artemisinin partial resistance in Africa: how do we respond? Lancet Infect Dis. 2024;24(9):e591–600. 38552654 10.1016/S1473-3099(24)00141-5 PMC12954456

[90] Mihreteab S, Anderson K, Molina-de la Fuente I, Sutherland CJ, Smith D, Cunningham J, Beshir KB, Cheng Q. The spread of a validated molecular marker of artemisinin partial resistance *pfkelch*13 R622I and association with *pfhrp*2/3 deletions in Eritrea. medRxiv. 2023: p. 2023.10. 20.23297302.

[91] Eloff L, Aranda-Díaz A, Routledge I, Wesolowski A, Chisenga M, Mangena B, Chimumbwa J, Sikaala C, Uusiku P, Katokele S. High prevalence of molecular markers associated with artemisinin, sulphadoxine and pyrimethamine resistance in northern Namibia*.* medRxiv. 2025: p. 2025.01. 09.25320247.10.4269/ajtmh.24-087040744004

[92] Meier-Scherling CP, Watson OJ, Asua V, Ghinai I, Katairo T, Garg S, Conrad M, Rosenthal PJ, Okell LC, Bailey JA. Selection of artemisinin partial resistance *Kelch*13 mutations in Uganda in 2016–22 was at a rate comparable to that seen previously in South-East Asia*.* medRxiv. 2024.

[93] Martin AC, Sadler JM, Simkin A, Musonda M, Katowa B, Matoba J, Schue J, Simulundu E, Bailey JA, Moss WJ. Emergence and rising prevalence of artemisinin partial resistance marker *Kelch*13 P441L in a low malaria transmission setting in Southern Zambia. J Infect Dis. 2025: p. jiaf188. 10.1093/infdis/jiaf188 PMC12526945 40241390

[94] Onchieku NM, Gesusu N, Caspar E, Karani L, Thiong’o K, Kamau L, Kiboi D, Thiebaut L, Ma L, Kimani F. Prevalence of Plasmodium falciparum parasites harbouring chloroquine-resistant but not artemisinin-resistant alleles in Busia County, Western Kenya. Malar J. 2025;24(1):247. 40739505 10.1186/s12936-025-05486-4 PMC12312427

[95] Akala H, Opot B, Juma DW, Okoth RO, Mwalo M, Salim FA, Maisiba R, Yedah RA, Mwakio EW, Chemwor G. Detection of twenty-four *Plasmodium falciparum Kelch* 13 mutations including C469Y, P553L, R561H, and A675V across Kenya. 2024.

[96] Kayode T, Niyukuri D, Holzschuh A, Da Silva G, Huwe T, Lerch A, Nyandwi J, Koepfli C. A novel* Plasmodium falciparum Kelch13 A675T *mutation and high levels of chloroquine and sulfadoxine-pyrimethamine resistance in burundi. medRxiv, 2025: p. 2025.06. 22.25330092.

[97] João MF, Aranda-Díaz A, De Amaral F, Makhanthisa TI, Lauterbach SB, Chisenga M, Mangena B, Maquina P, Routledge I, Sikaala C. Geographical heterogeneity in antimalarial resistance markers revealed by genomic surveillance in Angola. 2023. medRxiv. 2025.10.4269/ajtmh.25-022641843937

[98] Gashema P, Kagame J, Iradukunda PG, Siddig EE, Tessema SK, Tegegne MA, Mazaba ML, Fallah M, Ngamije D, de Dieu Harelimana J. Mapping *Plasmodium falciparum* mutations in Africa: a critical review of emerging drug resistance and implications for malaria control. Int J Infect Dis, 2025:108033. 10.1016/j.ijid.2025.108033 PMC12485076 40885517

[99] Zhao H, Pi L, Zhao L, Qin Y, Zeng W, Xiang Z, Yang Q, Pan M, Li X, Zou C. First detection in West Africa of a mutation that may contribute to artemisinin resistance Plasmodium falciparum. Front Genet. 2021;12:701750. 34691144 10.3389/fgene.2021.701750 PMC8531651

[100] Aranda-Díaz A, Mwanza S, Makhanthisa TI, Lauterbach SB, De Amaral F, Chisenga M, Mangena B, Routledge I, Letinić B, Kasonde B. *Plasmodium falciparum* genomic surveillance reveals a diversity of *kelch13* mutations in Zambia. medRxiv. 2025: p. 2025.02. 19.25322554.10.4269/ajtmh.25-011040744006

[101] Kahunu GM, Thomsen SW, Thomsen LW, Mavoko HM, Mulopo PM, Hocke EF, Nkoli PM, Baraka V, Minja DT, Mousa A. Identification of the PfK13 mutations R561H and P441L in the Democratic Republic of Congo. Int J Infect Dis. 2024;139:41–9. 38016502 10.1016/j.ijid.2023.11.026

[102] Schreidah C, Giesbrecht D, Gashema P, Young NW, Munyaneza T, Muvunyi CM, Thwai K, Mazarati J-B, Bailey JA, Juliano JJ. Expansion of artemisinin partial resistance mutations and lack of histidine rich protein-2 and-3 deletions in Plasmodium falciparum infections from Rukara, Rwanda. Malar J. 2024;23(1):150. 38755607 10.1186/s12936-024-04981-4 PMC11100144

